# Estradiol Downregulates MicroRNA-193a to Mediate Its Anti-Mitogenic Actions on Human Coronary Artery Smooth Muscle Cell Growth

**DOI:** 10.3390/cells14151132

**Published:** 2025-07-23

**Authors:** Lisa Rigassi, Marinella Rosselli, Brigitte Leeners, Mirel Adrian Popa, Raghvendra Krishna Dubey

**Affiliations:** 1 Department of Obstetrics and Gynaecology, Clinic for Reproductive Endocrinology, University Hospital Zurich, 8952 Schlieren, Switzerlandmarinella.rosselli@usz.ch (M.R.); brigitte.leeners@usz.ch (B.L.); 2 Institute of Cellular Biology and Pathology “Nicolae Simionescu” of the Romanian Academy, 050568 Bucharest, Romania; mirel.popa@icbp.ro; 3 Department of Pharmacology & Chemical Biology, University of Pittsburgh, Pittsburgh, PA 15219, USA

**Keywords:** estradiol, miRNA-139a, smooth muscle cells, proliferation, migration, cardiovascular disorders

## Abstract

The abnormal growth of smooth muscle cells (SMCs) contributes to the vascular remodeling associated with coronary artery disease, a leading cause of death in women. Estradiol (E2) mediates cardiovascular protective actions, in part, by inhibiting the abnormal growth (proliferation and migration) of SMCs through various mechanism. Since microRNAs (miRNAs) play a major role in regulating cell growth and vascular remodeling, we hypothesize that miRNAs may mediate the protective actions of E2. Following preliminary leads from E2-regulated miRNAs, we found that platelet-derived growth factor (PDGF)-BB-induced miR-193a in SMCs is downregulated by E2 via estrogen receptor (ER)α, but not the ERβ or G-protein-coupled estrogen receptor (GPER). Importantly, miR-193a is actively involved in regulating SMC functions. The ectopic expression of miR-193a induced vascular SMC proliferation and migration, while its suppression with antimir abrogated PDGF-BB-induced growth, effects that were similar to E2. Importantly, the restoration of miR-193a abrogated the anti-mitogenic actions of E2 on PDGF-BB-induced growth, suggesting a key role of miR-193a in mediating the growth inhibitory actions of E2 in vascular SMCs. E2-abrogated PDGF-BB, but not miR-193a, induced SMC growth, suggesting that E2 blocks the PDGF-BB-induced miR-193a formation to mediate its anti-mitogenic actions. Interestingly, the PDGF-BB-induced miR-193a formation in SMCs was also abrogated by 2-methoxyestradiol (2ME), an endogenous E2 metabolite that inhibits SMC growth via an ER-independent mechanism. Furthermore, we found that miR-193a induces SMC growth by activating the phosphatidylinositol 3-kinases (PI3K)/Akt signaling pathway and promoting the G1 to S phase progression of the cell cycle, by inducing Cyclin D1, Cyclin Dependent Kinase 4 (CDK4), Cyclin E, and proliferating-cell-nuclear-antigen (PCNA) expression and Retinoblastoma-protein (RB) phosphorylation. Importantly, in mice, treatment with miR-193a antimir, but not its control, prevented cuff-induced vascular remodeling and significantly reducing the vessel-wall-to-lumen ratio in animal models. Taken together, our findings provide the first evidence that miR-193a promotes SMC proliferation and migration and may play a key role in PDGF-BB-induced vascular remodeling/occlusion. Importantly, E2 prevents PDGF-BB-induced SMC growth by downregulating miR-193a formation in SMCs. Since, miR-193a antimir prevents SMC growth as well as cuff-induced vascular remodeling, it may represent a promising therapeutic molecule against cardiovascular disease.

## 1. Introduction

Estrogens play a crucial role in the regulation of various physiological processes in the body, including the cardiovascular system. Epidemiologic studies indicate estrogens’ protective effects against cardiovascular disease (CVD). Indeed, the risk of CVD is generally lower in woman compared to age-matched men and increases after the onset of menopause, as indicated by the increased intimal thickening and plaque formation [1]. Moreover women undergoing hormone replacement therapy have a lower risk of coronary disease [2,3] and overall mortality [4].

The beneficial effects of estrogens like estradiol (E2) in preventing pathological processes associated with the development of hypertension, thrombosis, restenosis, cardiomyopathy, atherosclerosis, and other CVDs have been confirmed by numerous studies [5]. E2 plays a crucial role in maintaining vascular health through various mechanisms, including the modulation of lipid profiles [6], improvement in endothelial function [7], and reduction in inflammatory responses [8]. E2 positively influences endothelial cell (EC) proliferation and migration, thereby inducing re-endothelization. Moreover, E2 exerts its protective effects by modulating vascular smooth muscle cells (SMCs), which are essential for the structural integrity and function of blood vessels [9]. E2 has been shown to inhibit the proliferation and migration of vascular SMCs, by various mechanisms. By binding to estrogen receptors (ERs) on vascular SMCs, E2 activates signaling pathways that lead to the downregulation of growth factors and cytokines involved in cell proliferation and migration. Furthermore, there is evidence that cell cycle progression is arrested by E2 metabolites such as 2-Methoxyestradiol (2ME) [10,11]. Recent studies also suggest microRNAs (miRNAs) as a new regulatory mechanism of estrogen vascular action [12].

Micro-RNAs are small, endogenous non-coding RNAs that influence the regulation of gene expression by targeting messenger RNAs (mRNAs) for degradation or translational repression, thus modulating many biological processes, including proliferation and migration, and in playing a crucial role in the pathophysiology of several diseases, including cardiovascular disorders [13,14,15,16,17]. Indeed, many miRNAs are dysregulated in diseased vessels, hence providing evidence of a key role in vascular remodeling associated with CVD [18,19].

E2 has been shown to modulate the expression of various miRNAs in different human cell lines and tissues [20], thereby influencing numerous cellular processes, including those related to cardiovascular health [21]. Moreover, recent findings provide evidence that miRNAs may play an important role in meditating the protective effects of E2 in vascular cells [12]. For instance, E2 has been shown to upregulate miRNAs that promote EC proliferation and migration, such as miR-21 and miR-126, which enhance angiogenesis and vascular repair by targeting specific genes involved in cell cycle regulation and migration pathways [22,23]. Moreover, E2 can modulate miRNAs that are associated with inflammation and atherosclerosis, such as miR-21 and miR-146a [24,25]. In vascular SMCs, the ER-mediated regulation of miR-203 inhibits proliferation [26]. Additionally, E2 regulates different miRNAs involved in vascular SMC growth [17], for example, the miR-124 [27,28] and miR-143/145 cluster [29,30]. Recent findings from our lab showed that miR-193a is differentially regulated by E2 and 2ME in vascular endothelial progenitor cells (EPCs; our unpublished data) and that it mediates the protective effects of E2 in vascular ECs [31]

The abnormal growth of SMCs plays a key role in vascular remodeling associated with occlusive disorders including coronary artery disease, atherosclerosis, and pressure- or injury-induced intimal thickening [32]. Although several growth factors generated locally or within the circulation have been implicated in the remodeling process, the platelet-derived growth factor (PDGF) is the most important [33,34]. Indeed, the upregulation of PDGF-BB has been shown to be responsible for neo-intimal thickening in both endothelial injury and non-injury (cuff-induced) vascular remodeling [35]. Since growth factors are known to modulate miRNAs in vascular cells including SMCs [36,37], it is feasible that they actively involved in mediating or regulating their mitogenic actions in SMCs. Indeed, miRNAs per se have been shown to influence SMC growth [17]. Since PDGF-BB is a potent mitogen for SMCs and involved in vascular remodeling; E2 is known to inhibit PDGF-BB-induced SMC growth; miRNAs can modulate SMC growth; and E2 modulates miR-193a to mediate its mitogenic actions in ECs [38], we hypothesize that estrogen may also influence vascular SMC growth and migration by downregulating miR-193a.

To test our hypothesis, we first tested the influence of PDGF-BB on various miRNAs that were modulated by E2 in EPCs (our unpublished data). Subsequently, we selected miR-193-3p to assess, in-depth, its role in E2-mediated growth inhibitory actions on SMCs and vascular remodeling. Using cultured human coronary artery SMCs, and scratch and proliferation assays, as well as a Western blotting of cell cycle and RT-PCR, we tested whether miR-193-3p mediates the mitogenic actions of PDGF-BB in SMCs at the cellular (cell growth) and molecular level (signal transduction and cell cycle regulation); E2 inhibits PDGF-BB-induced mitogenic actions by modulating miR-193a; ERα, ERβ, or G-protein-coupled estrogen receptor (GPER) are involved in mediating the modulatory effects of E2 on PDGF-BB-induced miR-193a; E2 counteracts the direct mitogenic actions of miR-193a in SMCs; E2 targets key cell-cycle pathways (positive and negative regulators of cell growth-Cyclin D1 and p27, respectively) triggered by PDGF-BB via miR-193a; and miR-193a antimir can prevent cuff-induced vascular remodeling/occlusion.

## 2. Materials and Methods

### 2.1. Cell Culture

Human coronary artery SMCs (SMCs, Life Technologies, Carlsbad, CA, USA; C-017-5C) between 4th and 8th passage were cultured in M231 culture medium (Life Technologies, CA, USA; M-231-500) supplemented with antibiotic-antimycotic (AA, Life Technologies, Carlsbad, CA, USA; 15240-096) and SMGS (Life Technologies, CA, USA; S-007-25). Cells were cultured in 75 cm^2^ flasks (TPP, Trasadingen, Switzerland) under standard tissue culture conditions (37 °C, 5% CO_2_). Upon confluence, cells were washed twice with HBSS -Ca^2+^/-Mg^2+^ (Bioconcept, Allschwil, CH; 3-02K34-I) and incubated for 3 min at 37 °C with trypsin (Sigma-Aldrich, St. Louis, MO, USA; T-3924) diluted 1 to 4 in HBSS -Ca^2+^/-Mg^2+^. An equal amount of growing medium was added to neutralize the reaction prior to 5 min centrifugation at 1200 rpm. Cells were resuspended in fresh media and either plated in new 75 cm^2^ flasks at a rate of 1:4 or seeded onto tissue culture dishes or multiwall plates (BD, Franklin Lakes, NJ, USA).

### 2.2. Transfection of SMCs with miRNA-193a Mimic and Antimir

SMCs were plated in growing medium and allowed to recover at least for 24 h. Cells were transfected with has-miRNA-193a.3p (mature sequence 5′-AAC UGG CCU ACA AAG UCC CAG U-3′) AccuTarget microRNA Mimic or Antimir (Bioneer, CA, USA; SMM-001 and SMI-001) and their respective negative control (AccuTarget Negative controls Mimic or Antimir, Bioneer, Oakland, CA, USA; SMC-2001 and SMC-2101). Transfection was performed using Lipofectamine2000 (Life Technologies, Carlsbad, CA, USA; 11668019). Briefly, the appropriate amount of Lipofectamine2000 was diluted in serum- and antibiotic-free DMEM/F12 in one tube (solution A), while miRNA oligonucleotides were diluted with the same media in a different tube (solution B). The solutions were incubated 5 min at room temperature before being equally mixed (A + B) and incubated 20 min at room temperature to allow the miRNA olignonucleotides-Lipofectamine2000 complexes to form. The cells were rinsed with serum- and antibiotic-free DMEM/F12 and the miRNA oligonucleotides-Lipofectamine2000 complexes added to each well, at a final Mimic or Antimir concentration of 25 nM. Mock transfection was carried out as described above but with the omission of the miRNA oligonucleotides. The transfection media were removed 5–6 h post transfection and either replaced by the respective regular culture media or treatments.

### 2.3. Transfection Efficiency

Transfection efficiency was determined using miRIDIAN Control Mimic and Antimir labeled with Dy547 (Dharmacon, Lafayette, CO, USA; CP-004500-01-05 and IP-004500-01-05). The transfected cells were stained with 0.5 ug/mL Hoechst33342 (Life Technologies, Carlsbad, CA, USA; H3570) and incubated 30 min prior to detection under a fluorescence microscope (Olympus, Volketswil, Switzerland). Quantitation of transfection efficiency was achieved by employing a GUAVA easyCyte HT Flow Cytometer (Merk Millipore, Schaffhausen, Switzerland). Transfected cells were detached by trypsinization, and centrifuged and resuspended in 300 µL sample buffer (1 g/L glucose in PBS) before acquisition of the fluorescent signal. Cells transfected with unlabeled Mimic and Antimir were used as negative controls.

### 2.4. Expression of miRNA-193a by qRT-PCR

For expression studies, SMCs were treated for 24 h by 20 ng/mL PDGF-BB (Sigma-Aldrich, St. Louis, MO, USA; P-3201) in the presence or absence of 10 and 100 nM 17-β-Estradiol (E2, Steraloids, Newport, RI, USA; E950). DMSO (Sigma-Aldrich, St. Louis, MO, USA; D2650) at a final concentration of 0.1% was applied as a vehicle-treated control.

The role of ERs was investigated using ER agonists PPT (Tocris, Bristol, UK; 1426), DPN (Tocris, Bristol, UK; 1494) and G1 (Calbiochem, Darmstadt, Germany 371705) at 100 nM each. ERα antagonists MPP (500 nM, Tocris, Bristol, UK; 1991) and ICI 182-780 (ICI, 1 µM, Tocris, Bristol, UK; 1047) were added 1 h prior to E2 or PPT. To study the role of estrogen metabolites, the cells were treated with 0, 1, 3, and 5 µM 2-Methoxyestradiol (2ME, Steraloids, Newport, RI, USA) during 24 h, prior to RNA extraction and miR-193a expression determination by qRT-PCR.

MiRNA-193a overexpression and downregulation were assessed 24 h, 48 h, and 72 h after transfection with the Mimic, the Antimir, and their respective negative controls. For pri-miRNA expression analysis, the cells were stimulated for 6 and 24 h with or without 10 nM E2 in starving media. Total RNA, including small RNAs, was extracted using the Quick-RNA MiniPrep Kit (ZymoResearch, Irvine, CA, USA; R1055), according to the manufacture’s protocol, and quantifies by the absorbance at 260 nm using the Infinite200 NanoQuant (Tecan, Männedorf, Switzerland).

RT-PCR for miRNA-193a was performed using a TaqMan microRNA assay (Life Technologies, Carlsbad, CA, USA; 4440887, Assay ID 000524, mature sequence 5′-AAC UGG CCU ACA AAG UCC CAG U-3′) which provides miRNA-specific RT primers as well as primers and probes for amplification and detection of the miRNA. Briefly, single-stranded cDNA was synthetized from 10 ng total RNA in 15 μL reaction volume with the TaqMan microRNA Reverse-Transcription Kit (Life Technologies, Carlsbad, CA, USA; 4366597). Each 15 μL reaction contained 1 mM dNTP mix, 50 U MultiScribe Reverse Transcriptase, 1× Reverse Transcription Buffer, 0.3 U RNase Inhibitor, and 1× microRNA-specific RT primers. The reaction was incubated at 16 °C for 30 min, followed by 30 min at 42 °C, and inactivation at 85 °C for 5 min. Samples were chilled on ice and diluted by addition of 75 μL DEPC-treated water (Life Technologies, Carlsbad, CA, USA; AM9916). Amplification and detection of the specific products were performed on a Bio-Rad CFX Real-Time PCR Detection System (Biorad, Reinach, Switzerland). PCR reaction included 5 μL 2× TaqMan Fast Advanced Master mix (Life Technologies, Carlsbad, CA, USA; 4444964), 0.5 μL each 20× TaqMan microRNA Assay mix, 0.5 μL DEPC-treated water, and 4 μL cDNA. The PCR reaction plate was run as follows: 2 min at 50 °C and 20 s at 95 °C, followed by 40 cycles of 95 °C for 3 s and 60 °C for 30 s. As internal controls, U48 (Life Technologies, Carlsbad, CA, USA; 4440887, Assay ID 001006, mature sequence 5′-GAT GAC CCC AGG TAA CTC TGA GTG TGT CGC TGA TGC CAT CAC CGC AGC GCT CTG ACC-3′) and U49 (Life Technologies, Carlsbad, CA, USA; 4440887, Assay ID 001005, mature sequence 5′-CAC TAA TAG GAA GTG CCG TCA GAA CGA TAA CTG ACG AAG ACT ACT CCT GTC TGA TT-3′) were used for miRNA template normalization.

For pri-miRNA expression, the TaqMan High-Capacity cDNA Reverse-Transcription Kit (Life Technologies, Carlsbad, CA, USA; 4427012, Assay ID Hs03303307_pri) was used to synthetize single-stranded cDNA from the extracted RNA. Briefly, 20 μL reaction contained 1× Reverse Transcription Buffer, 1× RT Random Primers, 4 mM deoxynucleotide (dNTP) Mix, 50 U MultiScribe Reverse Transcriptase, nuclease-free water, and 10 µL of 20 μg/μL RNA. The reaction mixture was incubated at 25 °C for 10 min, followed by 120 min at 37 °C and inactivation at 85 °C for 5 min. cDNA was stored at −20 °C or placed on ice for immediate procession with qPCR. Amplification and detection of the specific products were performed on a Bio-Rad CFX Real-Time PCR Detection System. PCR reaction included 5 μL 2× TaqMan Fast Advanced Master mix, 0.5 μL each 20× TaqMan pri-miRNA Assay mix, 2.5 μL DEPC-treated water, and 2 μL cDNA. The PCR reaction plate was run as follows: 2 min at 50 °C and 20 s at 95 °C, followed by 40 cycles of 95 °C for 3 s and 60 °C for 30 s. As an internal control, GAPDH (Life Technologies, Carlsbad, CA, USA; 4331182, Assay ID Hs99999905_m1) and hPRT1 (Life Technologies, Carlsbad, CA, USA; 4331182, Assay ID Hs99999909_m1) were used for pri-miRNA template normalization.

The relative gene expression of pri- and mature miRNA was assessed by comparing cycle times (Ct) through the CFX Connect software version 1.4.1 (Biorad, Reinach, Switzerland). Target Ct values were normalized by subtracting the internal control values, which provided the ΔCt value. The relative expression level between treatments was calculated using the following equation: 2^−(ΔCt sample − ΔCt control).^

### 2.5. SMC Growth Studies: Proliferation and Migration

SMCs were stimulated with 20 ng/mL PDGF-BB in starving media and treated with 10 and 100 nM E2. SMC proliferation was determined by cell counting and bromodeoxyuridine (BrdU) incorporation assay. For cell counting, cells were plated in 12-well plates and growth arrested in starving media for 24 h before treatment. For the investigation of miRNA-193a role, cells were starved overnight prior to cell transfection and treated 5–6 h post transfection with or without PDGF-BB and E2. After three days, cells were dislodged by trypsinization and counted in a Coulter Counter. For BrdU-incorporation assay, the chemiluminescent Cell Proliferation ELISA Kit (Roche, Switzerland; 11669915001) was used, according to manufacturer’s protocol. Then 5000 cells/well were plated on a black 96-well plate and allowed to attach overnight. The next day, the cells were treated or transfected with Mimics and Antimirs prior to treatment. Then, 10 μM BrdU was added after 24 h and allowed to incorporate into the cells for additional 24 h. Cells were fixed and stained with anti-BrdU antibody, and the chemiluminescent signal measured with a Infinite200 microplate reader (Tecan, Salzburg, Austria).

SMC migration was determined by a scratch wound assay. SMCs at 100% confluence in 6-well plates were wounded with a sterile pipette to generate a cell-free gap. The cells were then washed twice with PBS and photographed before treatment at 4× magnification using a bright field microscope (Olympus, Switzerland), to record the wound width after the scratch (T0). For the investigation of miRNA-193a’s role, cells were grown to 80% confluence before transfection and allowed to grow to confluence 48 h post transfection, before scratching and treatment. Then, 24 h later (T24), photographs were taken again. Wound closure was determined using Xcellence Pro software v1.2 (Olympus, Switzerland): wound area at T0 was subtracted by wound area at T24 and divided by wound area at T0 to normalize for the differences in wound size.

### 2.6. Protein Expression Studies

For protein expression and phosphorylation analysis in SMCs, cells were serum-starved for 24 h and treated for 48 h in presence or absence of 20 ng/mL PDGF-BB and 10–100 nM E2. Mimic- and Antimir-transfected cells were serum-starved overnight prior to transfection and treated for 48 h in starving medium or 20 ng/mL PDGF-BB post transfection. Mimic- and Antimir-transfected cells were kept in growing media for 48 h post transfection and stimulated for 45 min with or without 10 nM E2.

To test transfection efficiency, SMCs were lysed 24 h, 48 h, and 72 h after transfection with the GAPDH-targeting AccuTarget microRNA Mimic Positive Control (Bioneer, Oakland, CA, USA; SMC-1001). Cells were washed with cold PBS, lysed in 60 μL Lysis Buffer (Cell Signaling Tech., Danvers, MA, USA; 9803), and homogenized for 2–10 s by sonication. Protein concentration was determined using the BCA Protein Assay Kit (Pierce Biotechnologies, Rockford, IL, USA; 23227) according to the manufacturer’s protocol. Equal number of proteins were diluted with Lämmli Sample Buffer (4×, Biorad, Reinach, Switzerland; 161-0747) and DTT (20×, Fermantas, MD, USA; R0891), incubated for 5 min at 95 °C and subjected to SDS-PAGE. Standard Western blot analysis was conducted using anti-Akt (Cell Signalling Tech., Danvers, MA, USA; 9727), anti-phospho-Akt (Ser473; Cell Signalling Tech., Danvers, MA, USA; 9271), anti-CDK4 (Millipore, San Diego, CA, USA; 07-659), anti-Cyclin D1 (Millipore, San Diego, CA, USA; 06-137), anti-Cyclin E (Cell Signalling Tech., Danvers, MA, USA; 4129), anti-GAPDH (Abcam, Cambridge, UK; ab9484), anti-p27 (Millipore, CA, USA; 04-240), anti-PCNA (Millipore, San Diego, CA, USA; CBL407), and anti-pRB (BD biosciences, Franklin Lakes, NJ, USA; 554136) antibodies. Anti-β-actin antibody (Sigma-Aldrich, St. Louis, MO, USA; A5441) was used as a loading control.

### 2.7. Artery Cuff Experiments

The C57BL/6J mice used in this study were purchased from Jackson Laboratory (Stock No. 000664) and maintained at the “Nicolae Simionescu” Institute of Cell Biology and Pathology Animal Facility under specific pathogen-free conditions. The animals were housed in 12 h light and 12 h dark cycle. All experimental procedures involving animals were conducted in accordance with the European Union (EU) Directive 2010/63/EU [39] and approved by the national competent authority (Authorization No. 198 and 10 March 2022). For this study, C57BL/6J female mice were divided into four groups: Sham (n = 6), PBS + Pluronic^®^ F127 (Sigma Aldrich, St. Louis, MO, USA) (n = 6), miRNA193a-antimir Control + Pluronic^®^ F127 (n = 5), and miRNA193a-antimir + Pluronic^®^ F127 (n = 7). All animals were 12–18-weeks old, and weighed 17–27 g. On days 1, 7, 14, and 21, the antimir and control were given intravenously at a rate of 80 mg/kg. In addition, on day 1, the miRNA and controls were injected subcutaneously into Pluronic^®^ F127 at a dose of 80 µg/200 µL, following the protocol as outlined by Liu et al. [40].

The artery cuff experiment follows closely the experimental setup by Moroi et al. [41]. Briefly, mice were anesthetized with an intraperitoneal injection of Ketamine: 90 mg/kg and Xylazine: 10 mg/kg. The left femoral artery was isolated from surrounding tissues, loosely sheathed with a 2.0 mm polyethylene cuff made of PE-50 tubing (inner diameter, 0.56 mm; outer diameter, 0.965 mm; Becton Dickinson, Mountain View, CA, USA) and tied in place with an 6-0 suture. The cuff is larger than the vessel and does not obstruct blood flow. The right femoral artery was dissected from surrounding tissues (sham-operated), but a cuff was not placed. The femoral arteries were replaced, and the wounds were sutured. After recovery from anesthesia, the animals were given standard diet and water ad libitum. Tissue harvesting and histologic staining were performed. Then, 2 weeks after cuff placement, animals were anesthetized and sacrificed. Vessels were harvested and fixed with 4% paraformaldehyde (Sigma Aldrich, St. Louis, MO, USA). Both right and left femoral arteries were harvested. Each artery was embedded in paraffin, and cross-sections (7 mm) were continuously cut from one edge to the other edge of the cuffed portion, and in the corresponding segment of the contralateral control artery. Each section was mounted in order on five series of slides. Parallel sections were subjected to Masson–Goldner’s trichrome staining kit (Carl Roth GmbH, Karlsruhe, Germany). Morphometric analyses were performed on Masson–Goldner’s trichrome stained tissue. For each animal, 10 cross-sections from the cuffed left femoral artery and the control right femoral artery were photographed, and, for each artery section, the thickness of the intima and lumen were measured using a Carl Zeiss Vision AxioVision Viewer Vers 4.8 (Carl Zeiss, Oberkochen, Baden-Württemberg, Germany). For each artery section, the area of the Tunica Intima and Tunica Adventitia were measured. For area ratio calculations, four measurements were made using AxioVision analysis software: area inside the inner intima, and area the outer adventitia. The arterial sections morphometry and the intra-assay variability were accomplished using independent observers or by analyzing adjacent sections from the same vessel, and was <3 percent.

### 2.8. Statistical Analysis

Unless stated differently, all data are presented as mean ± standard error. Experiments were repeated at least three times. For statistical evaluation, Student’s *t* tests and ANOVA were used and statistical significance (*p* < 0.05) was calculated using Fisher’s Least Significant Difference test.

## 3. Results

### 3.1. Modulatory Effects of Estradiol on PDGF-BB-Regulated miRNAs in SMCs

#### 3.1.1. PDGF-BB-Regulated miRNAs in SMCs

**Figure 1:**
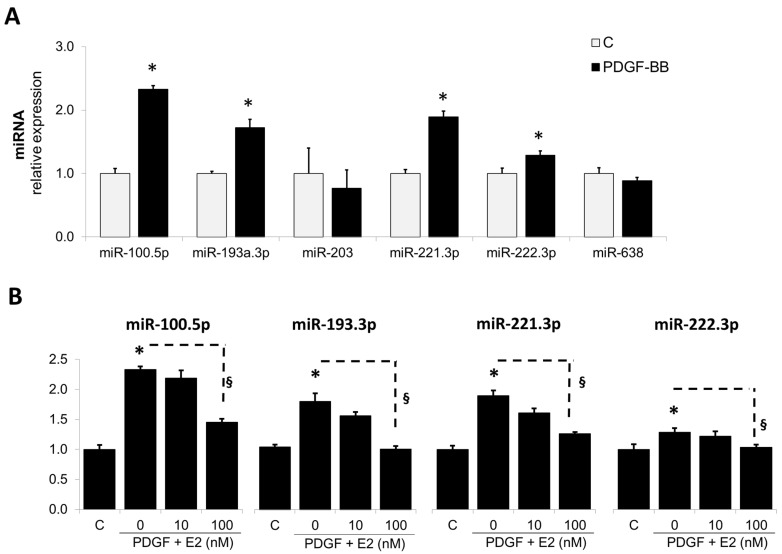
PDGF-BB regulates multiple miRNAs in SMCs and Estradiol (E2) downregulates PDGF-induced miR-193a expression in SMCs. Panel (**A**) depicts the modulatory actions of PDGF-BB on various miRNAs known to influence cell growth; Panel (**B**): depicts the concentration-dependent modulatory actions of E2 on PDGF-BB-induced miRNAs in SMCs. Cells were treated with PDGF-BB (20 ng/mL) and 0, 10, and 100 nM E2. After 24 h, RNA was extracted and miR-100-5p, miR-193a-3p, miR-203, miR-221-3p, miR-222-3p, miR-409-5p, and miR-638 levels determined by RT-qPCR. The results are presented as mean ± SEM (n = 4). * *p* < 0.05 versus control (C); ^§^ *p* < 0.05 between indicated treatments. MiRNA expression was normalized to U48 and U49.

The abnormal growth of SMCs contributes to the vascular remodeling process, leading to vascular occlusive disorders, and the role of PDGF-BB in driving these actions are well-established [42]. Since miRNAs are known to be biologically active and mediate pro-growth actions, we first assessed the modulatory effects of PDGF-BB on miRNAs known to influence cell growth. As shown in Figure 1A, the treatment differentially modulated the expression of various miRNAs. PDGF-BB upregulated the expression of miR-100-5p, miR-193-3p, miR-221-3p, and miR-222-3p, but not the expression of miR-203, miR-409-5p, and miR-638.

#### 3.1.2. Estradiol Abrogates the Stimulatory Effects of PDGF-BB on miRNA Expression

We have previously shown that E2 prevents vascular remodeling by abrogating the growth promoting effects of PDGF-BB on vascular SMCs [43]. Hence, to assess whether E2 may be mediating these actions by interfering with growth regulatory miRNAs, induced by PDGF, we assessed its impact on the expression of selected miRNAs. As shown in Figure 1B, E2 prevented the stimulatory effects of PDGF-BB on miR100.5p, miR-193-3p, miR221-3p, and miR222-p and significantly decreased their relative expression in response to PDGF-BB from 2.33 ± 0.05% to 1.45 ± 0.06%, from 1.73 ± 0.12% to 0.96 ± 0.05%, from 1.89 ± 0.09% to 1.26 ± 0.03%, and from 1.23 ± 0.05% to 1.03 ± 0.04%, respectively. Since miR-193a has been well-documented to influence vascular cell migration and proliferation by targeting growth-associated cell cycle mechanisms [44,45,46], we elected to study its involvement in mediating the protective (growth inhibitory) actions of E2 on vascular SMCs. As shown in Figure 1B, E2 inhibited the PDGF-BB-induced miR-193a expression in a concentration-dependent manner and fully abrogated its actions at 100 nM.

The biogenesis of miRNAs is regulated at multiple levels, including the modulation of the miRNA gene transcription procession to mature miRNAs by Drosha and Dicer in the nucleus and cytoplasm, respectively. Moreover, Argonaute RISC Catalytic Component 2 (AGO2) loading and RNA decay also modulate miRNA activity and expression [47]. Many miRNAs are regulated directly by transcription factors, as their promoter region is similar to that of regular protein coding genes. Since E2 interacts with its nuclear receptors and regulates gene expression including miRNA expression [12], we further investigated whether these effects were mediated at the transcriptional level. To achieve this goal, we examined the expression of pri-mRNAs following E2 treatment. Using RT-qPCR, we examined the expression of the pri-miRNAs in SMCs treated with E2.

**Figure 2:**
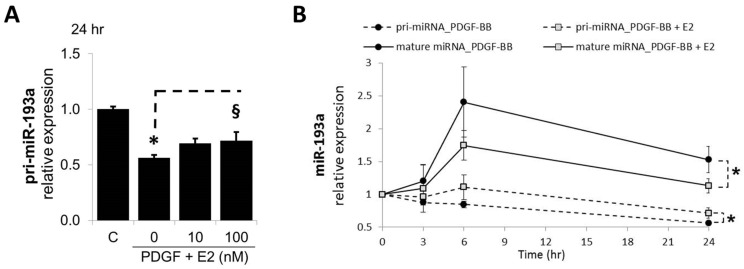
E2 Modulates miR-193a Processing in SMCs. Panel (**A**) depicts the concentration-dependent abrogatory effects of E2 on PDGF-BB-mediated decrease in pri-miR-193a. Cells grown to 60% confluency in complete media prior to treatment with PDGF-BB and E2 (0, 10, 100 nM). Total RNA was extracted after 24 h of treatment and relative pri-miR-193a levels determined by RT-qPCR using Taqman assays for pri-miR-193a. The results were normalized to Glyceraldehyde 3-phosphate dehydrogenase (GADPH) and Hypoxanthine-guanine phosphoribosyltransferase 1 (hPRT1) mRNAs (n = 3; * *p* < 0.05 compared to control; ^§^ *p* < 0.05 significant reversal of PDGF-BB effects). Panel (**B**) depicts the differential effects of E2 on pri-miR-193a and mature miR-193a in SMCs. Evidence that E2 abrogates the lowering effects of PDGF-BB on pri-miR-193a and the stimulatory effects on mature miR-193a in SMCs. Cells were treated for 0, 3, 6, and 24 h, and both pri- and mature miR-193a analyzed by RT-qPCR as above. The pri-miRNAs were normalized to GADPH and hPRT1 mRNAs, whereas the mature miRNA expression was normalized to U48 and U49.

In SMCs, the expression of the pri-miRNAs was strongly affected in SMCs treated for 24 h with PDGF-BB and this effect was significantly reversed by E2 (100 nM; Figure 2A). PDGF-BB reduced pri-miR-193a by 44 ± 2% (*p* < 0.05 versus untreated control, C); moreover, 10 and 100 nM of E2 abrogated the inhibitory effect of PDGF-BB in a concentration-dependent manner from 56 ± 2% to 69 ± 4% and 71 ± 8% (*p* < 0.05), respectively. Since the observed effects might be due to conversion of pri-miRNAs into mature miRNAs, we additionally looked at the time-dependent changes in pri-miRNA expression. A comparison of pri- and mature miR-193a after 3 and 6 h of treatment with E2 is shown in Figure 2B. Treatment with PDGF-BB decreased pri-miR-193a expression at 3, 6, and 24 h, while increasing mature miR-193a at the same time points. The co-treatment with E2 reversed the effects of PDGF-BB on both pri- and mature miR-193a at all the time points studied. Taken together, our findings provide evidence that E2 does not inhibit miR-193a transcription in SMCs, but most likely downregulates miR-193a by inhibiting their procession from the primary transcript to mature functional miRNAs. This contention is supported by the recent findings that E2–ER interaction plays a role in Drosha and Dicer function and that E2 regulates AGO2 [16,48].

### 3.2. miR-193-3p Transfection Efficiency in SMCs

**Figure 3:**
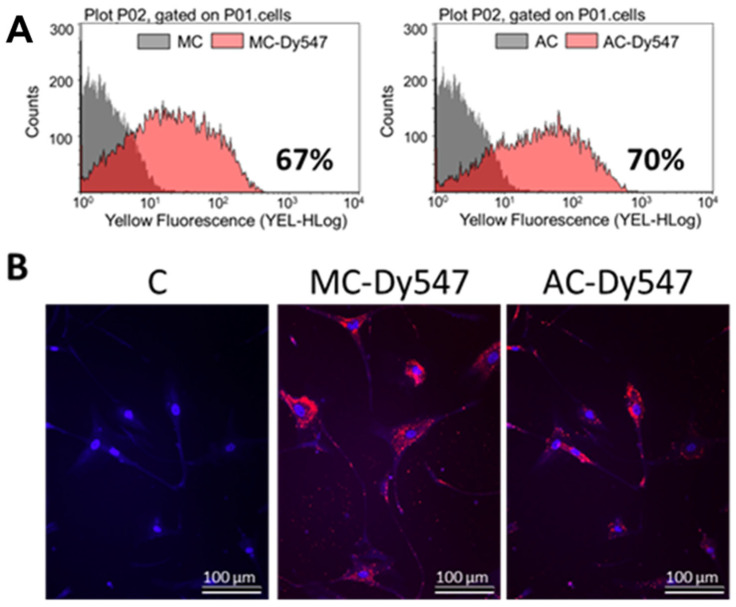
Transfection efficiency of miR-193a Mimic and Antimir in SMCs. Transfection efficiency was assessed using a 25 nM Dy547-labeled control Mimic (MC-Dy547) and Antimir (AC-Dy547). After 6 h transfection, using Lipofectamine 2000 in absence of antibiotics and serum, SMCs were allowed to recover for 24 h in growing medium. Panel (**A**) depicts flow cytometric profile of the percentage of Dy547-positive cells with respect to the control cells, transfected with unlabeled control Mimic (MC) and control Antimir (AC). Panel (**B**) shows representative fluorescent photomicrographs of SMCs transfected with MC and/or AC. Fluorescence: Red = Dy547-labeled Mimic or Antimir; blue = Hoechst33342 stain. Experiments were performed at least 3 times in triplicates.

To assess whether miR-193a mediates the growth regulatory actions of E2 in SMCs, we first established its transfection efficiency using a 25 nM Dy547-labeled control Mimic (MC-Dy547) and Antimir (AC-Dy547). After 6 h of transfection, using Lipofectamine 2000 in the absence of antibiotics and serum, SMCs were allowed to recover for 24 h in the growing medium. Flow cytometry was used to quantify the percentage of Dy547-positive cells with respect to the control cells, transfected with unlabeled control Mimic (MC) and control Antimir (AC) (depicted in Figure 3A), whereas fluorescence microscopy was used to take representative images (Red = Dy547-labeled Mimic or Antimir; blue = Hoechst33342 stain (Figure 3B)). Flowcytometeric data revealed a 67% transfection efficiency with MC and and 70% with AC. Moreover, this outcome was reflected by the positive staining of transfected SMCs.

**Figure 4:**
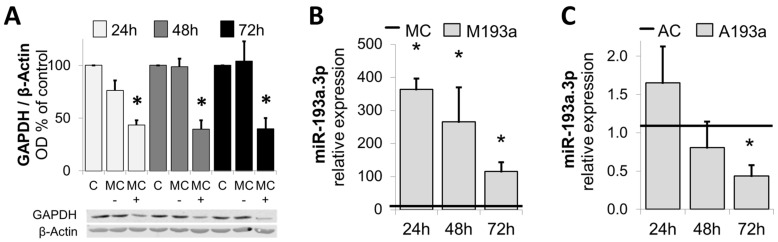
Confirmation of optimal transfection conditions: Panel (**A**): Western blots of lysates from SMCs transfected with 25 nM GAPDH-targeting Positive Control Mimic (MC+), Negative Control (MC−), or mock transfected (C) were kept in growing media for 24 h, 48 h, and 72 h prior to cell lysis and analysis of GAPDH protein expression using Western blot. β-Actin was used as a loading control. n = 3, * *p* < 0.05 compared to MC−. Panels (**B**,**C**) depict changes in miR-193a expression in SMCs by miR-193a Mimic (M) and Antimir (A), respectively. SMCs were transfected with 25 nM miR-193a Mimic (M193a, **B**) or Antimir (A193a, **C**) and the respective negative controls MC (Mimic) and AC (Antimir). RNA was extracted after 24 h, 48 h, and 72 h prior to determination of miR-193a level by RT-PCR. (n = 3). * *p* < 0.05 versus negative control (MC or AC).

After assessing the transfection efficiency of miR-193a in SMCs, we further assessed the optimal transfection experimental conditions by means of a positive control mimic specifically targeting Glyceraldehyde 3-phosphate dehydrogenase (GADPH). Figure 4A shows SMCs transfected with 25 nM GAPDH-targeting Positive Control Mimic (MC+), Negative Control (MC−), or mock transfected (C) were kept in growing media for 24 h, 48 h, and 72 h prior to cell lysis and analysis of GAPDH protein expression using Western blot. β-Actin was used as a loading control (n = 3, * *p* < 0.05 compared to MC−). The protein expression of GADPH was reduced by ≈40% (n.s.) 24 h post transfection and by ≈ 75% (*p* < 0.05) at 48 h and 72 h after transfection with the positive control mimic.

Next, we investigated the alteration in miR-193a expression by the ectopic application of miR-193a Mimic and Antimir. SMCs were transfected with 25 nM miR-193a Mimic (M193a) or Antimir (A193a) and the respective negative controls MC (Mimic) and AC (Antimir). RNA was extracted after 24 h, 48 h, and 72 h prior to the determination of the miR-193a level by RT-PCR. As shown in Figure 4B,C, compared to the mimic control (MC), miR-193a mimics significantly increased the levels of miR-193a in SMCs after 24 h, 48 h, and 72 h (Figure 4B)**.** Moreover, transfection with the Antimir 193a resulted in a time-dependent reduction in miR-193a levels (Figure 4C), which was significant after 72 h (32 ± 9.78% reduction; *p* < 0.05).

### 3.3. Ectopic Expression of miR-193a Stimulates SMC Growth and Migration

**Figure 5:**
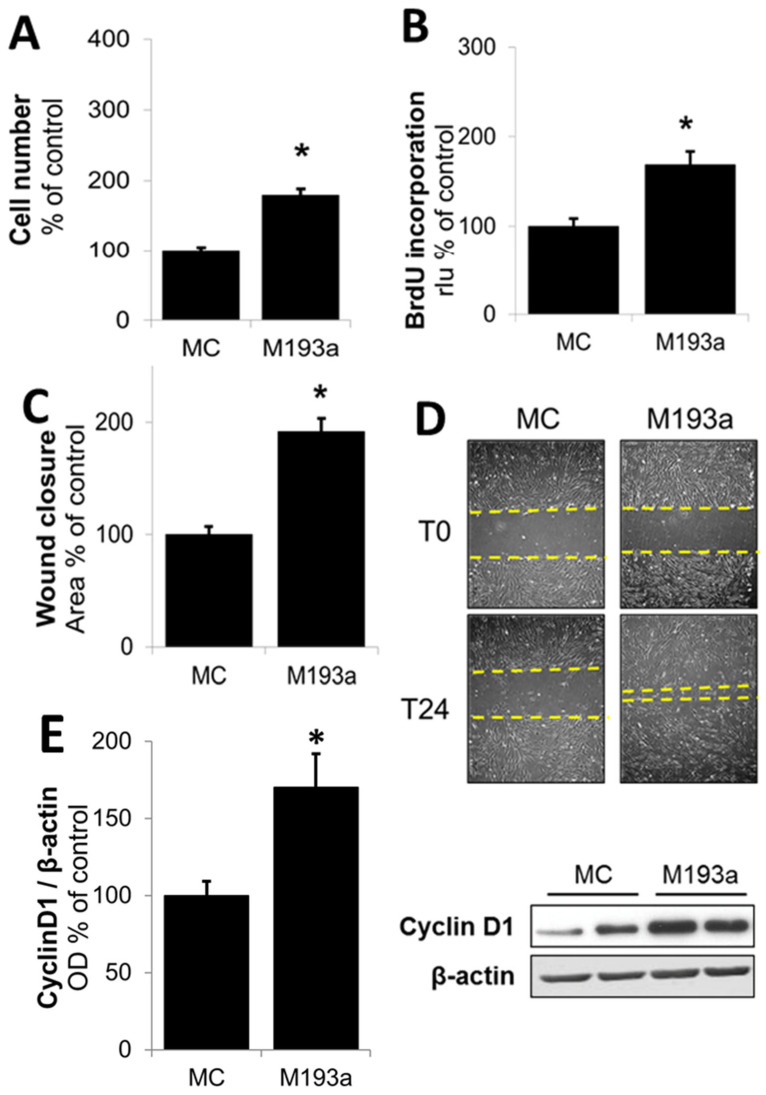
Ectopic expression of miR-193a stimulates SMC proliferation and migration. SMCs were transfected with 25 nM miR-193a Mimic (M193a) and control Mimic (MC). All the assays were made in serum-free media. Panel (**A)**: Cell number was assessed after 3D using a Coulter Counter. Panel (**B)**: DNA synthesis was determined using a BrdU-incorporation ELISA kit. Panels (**C**,**D**): SMC migration was determined using a scratch wound assay. For ectopic expression of miR-193a in SMCs, the cells were grown in complete media for 48 h after transfection, before the scratch was made and serum was removed. Representative images (**D**) for each condition are shown immediately after the scratch (T0) and 24 h later (T24) and marked by yellow dashed line. Panel (**E**): For Cyclin D1 expression, cells were starved 48 h after treatment/transfection prior to cell lysis and Western blot analysis. Bar graph depicts the change in the ratio of optical density (OD) between Cyclin D1 to β-actin. The results are presented as mean ± SEM (n = 3). * *p* < 0.05 versus the respective control (C or MC).

Following the optimization of miRNA transfection conditions, we further assessed the modulatory impact of miR-193a mimics (M193a) and antimirs (A193a) and their respective controls (MC and AC) on SMC growth (DNA synthesis and cell proliferation), migration (using wound closure assay), and changes in cell-growth-associated cell cycle driver Cyclin D1. As shown in Figure 5A,B, the transfection of SMCs with miR-193a significantly induced both the cell number and DNA synthesis (measured using BrdU incoporation). Compared to cells transfected with mimic control (MC), M193a induced cell number and DNA synthesis by 79 ± 8% (*p* < 0.05) and 69 ± 15% (*p* < 0.05), respectively. Moreover, transfection with M193a induced SMC migration in the scratch assay (Figure 5D,E). Compared to controls treated with MC, M193a induced cell migration by 92 ± 12% (*p* < 0.05). Since Cyclin D1 plays a key role in inducing PDGF-BB-induced growth in SMCs, we further assessed the impact of M193a on its expression. As shown in Figure 5E, Consistent with our observations on DNA synthesis, proliferation, and migration, transfection with 193a induced Cyclin D1 expression by 70 ± 22% (*p* < 0.05) as compared with cells transfected with MC (Figure 5E).

**Figure 6:**
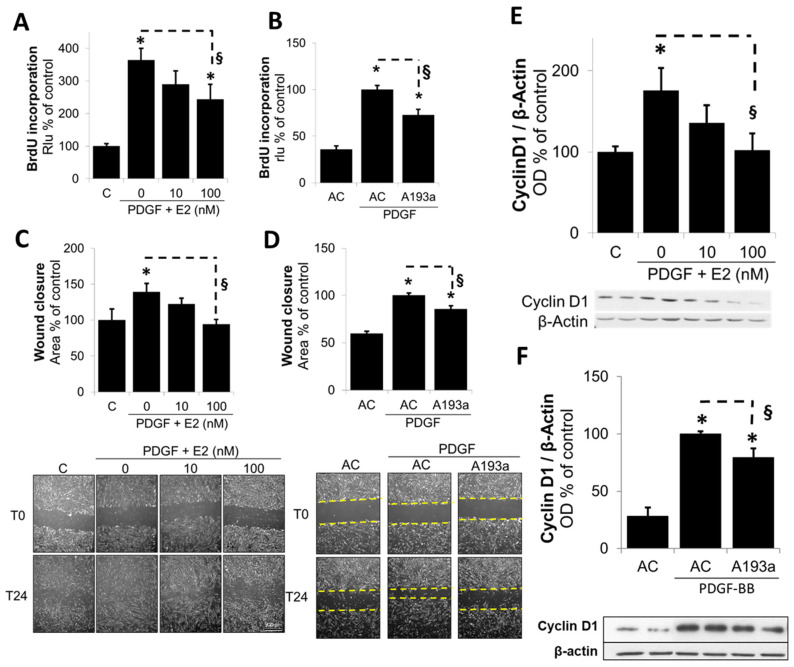
Ectopic expression of M193a Antimir mimics the effects of E2 and counteracts PDGF-BB-induced SMC growth. SMCs were either treated with 0, 10, and 100 nM E2 in presence of PDGF-BB (20 ng/mL) or transfected by 25 nM miR-193a Antimir (A193a) or control Antimir (AC) prior to PDGF-BB stimulation. Proliferation was measured by BrdU-incorporation assay (**A**,**B**); cell migration using scratch assay (**C**,**D**); and Cyclin D1 protein expression by Western blotting (**E**,**F**). SMCs were either treated with 0, 10, and 100 nM E2 in presence of PDGF-BB (20 ng/mL) or transfected by 25 nM miR-193a Antimir (A193a) or control Antimir (AC) prior to PDGF-BB stimulation. Panels (**C**,**D**) depict SMC migration as determined by using a 24 h scratch wound assay. Photomicrographs below the bar graphs depict representative images for each condition immediately after the scratch (T0) and 24 h later (T24) and marked with dashed line. The results are presented as mean ± SEM (n = 3). * *p* < 0.05 versus the respective control (C or AC without PDGF); ^§^ *p* < 0.05 between indicated treatments. Panels (**E**,**F**) depict bar graphs for PDGF-BB-induced Cyclin D1 expression in SMCs in presence of E2 (10 and 100 nmol/L E2; Panel (**E**) and SMCs transfected with miR-193a antimir (A193a) or antimir control (AC; Panel (**F**)). Representative blots for Cyclin D1 expression are presented below the bar graphs. The results are presented as mean ± SEM (n = 3). * *p* < 0.05 versus the respective control (C or AC without PDGF); ^§^ *p* < 0.05 between indicated treatments.

Since miR-193a mimicked the growth stimulatory effects of PDGF-BB on SMCs, we further confirmed this role by assessing whether the ectopic expression of its antimir A193a would counteract the effects of PDGF-BB on SMC growth. As shown in Figure 6, E2 inhibited PDGF-BB-induced DNA synthesis in a concentration-dependent fashion (Figure 6A); moreover, PDGF-BB-induced DNA synthesis was significantly inhibited in SMCs transfected with miR-193a antimir (A193a) as compred to cells transfected with antimir control (AC) (Figure 6B). These findings suggest that PDGF-BB induced SMC growth, in part by inducing miR-193a, and E2 blocks PDGF-BB-induced growth, in part, by downregulating the miR-193a expression.

### 3.4. Ectopic Expression of miR-193a Reverses the Protective Actions of E2 in SMCs

**Figure 7:**
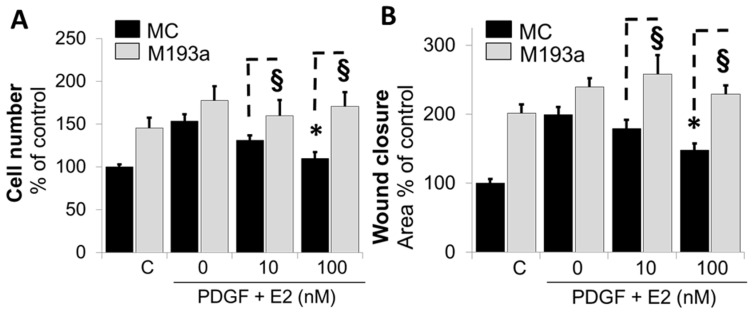
Ectopic expression of miR-193a in SMCs reverses the growth inhibitory actions of E2. SMCs were transfected with control and miR-193a mimic (MC and M193a) and treated post transfection with E2 (0, 10, and 100 nM) in presence of PDGF-BB (20 ng/mL). Panel (**A**): SMC proliferation was determined by counting the cells 3 days after treatment. Panel (**B**): For cell migration, SMCs were allowed to recover 48 h in growing media after transfection, before the scratch was made and the treatment added for 24 h. The results are presented as mean ± SEM (n = 3). * *p* < 0.05 versus SMCs treated with PDGF-BB alone or control (C). ^§^ *p* < 0.05 between indicated treatments.

Although our findings provide indirect evidence that miR-193a mimics the pro-growth actions of PDGF-BB and the inhibitory effects of E2 are mediated via the downregulation of miR-193a, direct evidence was lacking. To provide proof that E2 mediates its inhibitory actions on the PDGF-BB-induced growth and migration of SMCs, we further assessed whether miR-193a can counteract the inhibitory effects of E2 on PDGF-BB-induced SMC growth. As shown in Figure 7A,B, the treatment of SMCs transfected with mimic control (MC) with PDGF-BB induced cell number and cell migration, and this effect was reduced by E2 in a concentration-dependent fashion. Compared to mimic control (MC)-transfected cells, the inhibitory effects of E2 on cell growth and migration were significantly reversed in SMCs transfected with M193a. Compared to mimic control (MC), the ectopic expression of miR-193a (M193a) reversed the growth inhibitory effects of 100 nM E2 from 110 ± 7.4% to 170 ± 17% (*p* < 0.05, n = 3). Similarly, the ectopic expression of miR-193a reversed the inhibitory effects of 100 M E2 on PDGF-BB-induced wound closure from 148 ± 10% in MC-transfected cells to 229 ± 13% (*p*< 0.05, n = 3).

**Figure 8:**
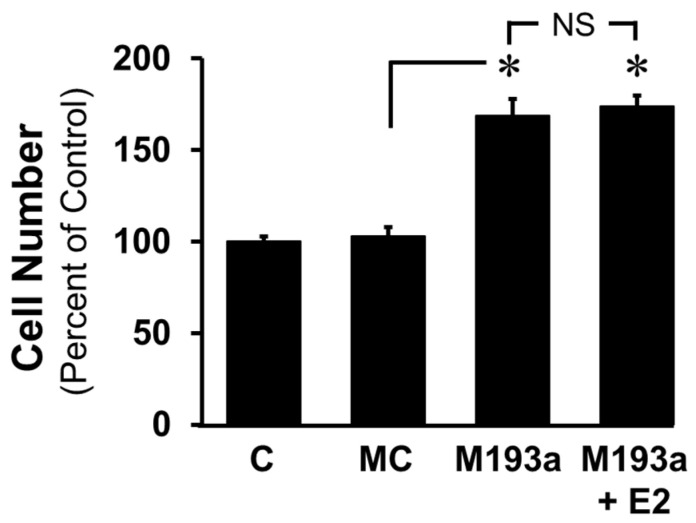
E2 does not block the mitogenic actions of miR-193a on SMC proliferation. SMCs were transfected with control and miR-193a Mimic (MC and M193a) and treated post transfection with E2 (100 nM). After 3 days, SMC proliferation was determined by counting the cells. The results are presented as mean ± SEM (n = 3). * *p* < 0.05 versus vehicle-treated control (C) and miRNA mimic control (MC). No significant difference (NS; *p* > 0.05) between the indicated treatments.

To further confirm whether E2 modulates the miR-193a-induced growth of SMCs, we assessed the effects of E2 (10 nM) on the miR-193a (M193a)-induced growth of SMCs. As shown in Figure 8, the ectopic expression of M193a but not mimic control (MC) induced SMC growth (cell number) and these effects were not blocked by E2. Collectively, the findings from Figure 7 and Figure 8 suggest that E2 selectively blocks the pro-growth effects of PDGF-BB by blocking miR-193-3p synthesis.

**Figure 9:**
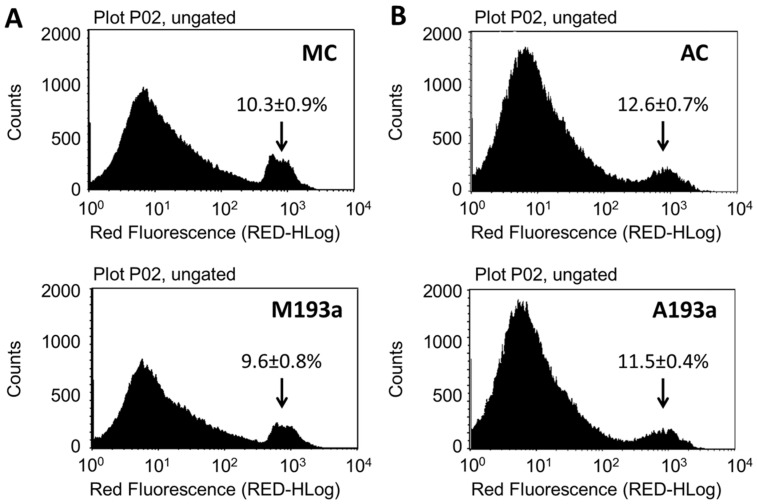
Alteration of miR-193a levels does not affect SMC viability. SMCs were transfected with Control Mimic (MC), miR-193a Mimic (M193a, Panel (**A**)), Control antimir (AC), and miR-193a antimir (A193a, Panel (**B**)), and cultured in growing media for 24 h. after transfection. For propidium iodide (PI) staining, the cells were trypsinized, incubated for 5 min with 0.2 µg/mL PI, and analyzed for viability with a flow cytometer. The arrow indicates percentage of dead cells. n = 3.

To make sure that the effects following transfection with miRNAs were not due to cell toxicity, the effects of both the miR-193a mimic (M193a) and antimir (A193a) were confirmed by assessing the cell viability using flow cytometry. As shown in Figure 9, we observed no significant loss in cell viability in SMCs transfected with M193a or A193a under our experimental conditions.

### 3.5. E2 Inhibits PDGF-Induced miR-193a Expression via ERα

**Figure 10:**
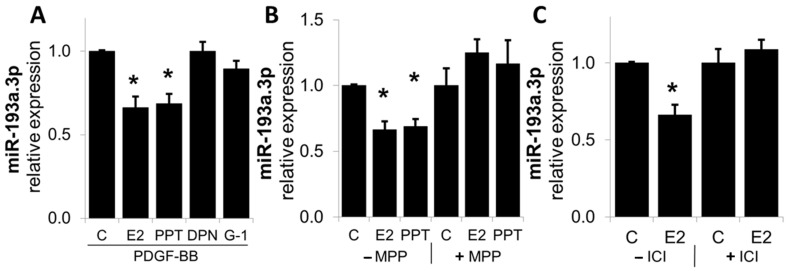
E2 modulated miR-193a expression via ER-α in SMCs. Panel (**A**): SMCs were treated with 100 nM E2 and ER agonists PPT (ER-α), DPN (ER-β), and G-1 (GPER) in presence of PDGF-BB (20 ng/mL) during 24 h, prior to RNA extraction and determination of miR-193a expression by qRT-PCR. Panel (**B**,**C**): Cells were pre-incubated for 1 h with the ER-α specific antagonist MPP (500 nM) and the unspecific ER antagonist ICI 182-780 (1 µM). The results are presented as mean ± SEM (n = 3). * *p* < 0.05 versus control.

Since estrogen mediates many of its cellular actions via ERα, ERβ, or GPER/GPR30, we assessed their role in mediating the actions of E2 on miR-193a expression in SMCs. As shown in Figure 10, the treatment of SMCs with ERα agonist PPT, but not ERβ and GPER agonists (DPN and G1, respectively), inhibited PDGF-BB-induced miR-193a expression in SMCs. Moreover, the modulatory effects of both E2 and PPT were blocked by the Erα-specific agonist MPP, as well as by ICI182780 (ICI), a non-specific ER antagonist. 

**Figure 11:**
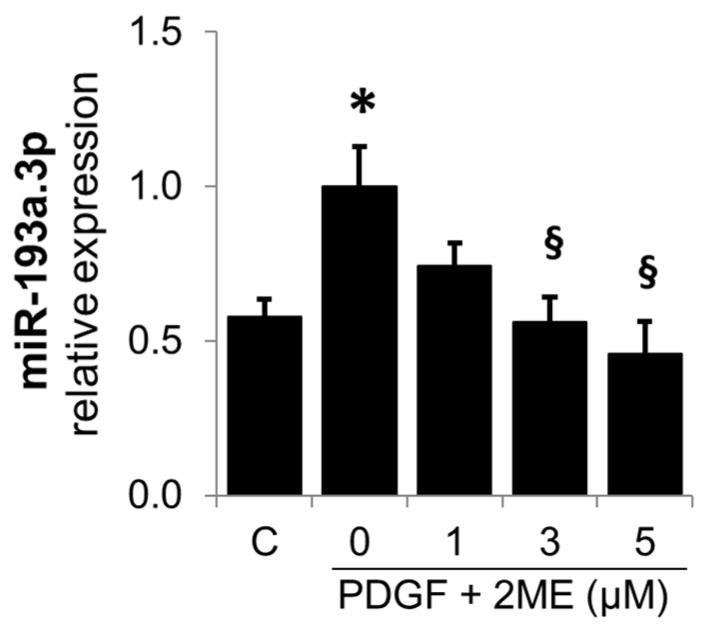
2ME, an endogenous downstream metabolite of E2, inhibits PDGF-BB-induced miR-193a.3p expression in SMCs. Cells were treated with 0 to 5 µM of 2ME in presence of PDGF-BB (20 ng/mL) during 24 h, prior to RNA extraction and miR-193a expression determination by qRT-PCR. The results are presented as mean ± SEM (n = 3). * *p* < 0.05 versus control, ^§^ *p* < 0.05 versus PDGF-BB treatment alone.

Since the downstream metabolites of E2 can also mediate the inhibitory actions of E2 on SMC growth [11], we also investigated its modulatory actions on the PDGF-BB-induced miR-193a.3p expression in SMCs. As shown in Figure 11, the treatment with 2ME inhibited miR-193a.3p expression, suggesting that E2 as well as its downstream metabolites may inhibit PDGF-BB-induced SMC growth by downregulating miR-193a.3p expression.

### 3.6. MiR-193a Mimics the Effects of PDGF-BB on Key SMC Growth Promoting Signal Transduction Activities and Cell Cycle Proteins

**Figure 12:**
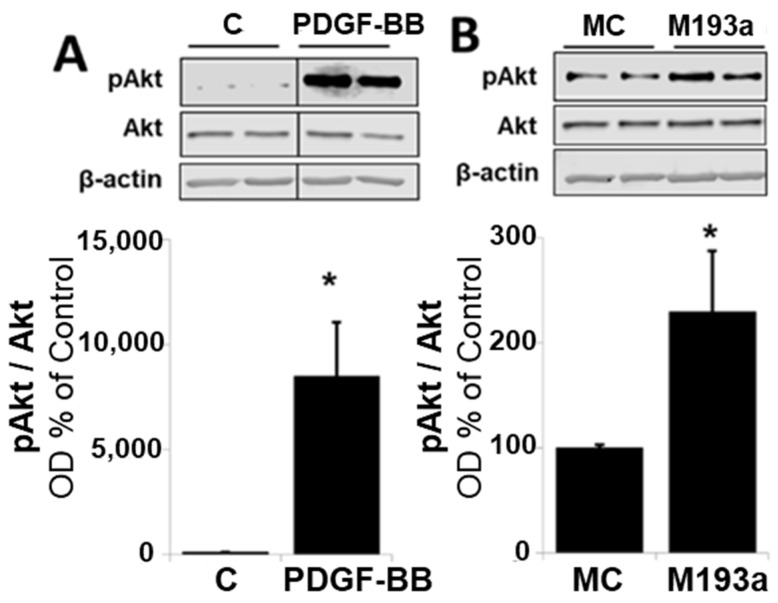
Overexpression of miR-193a promotes G1-to-S-phase progression. Representative Western blots and bar graphs of phosphorylated Akt (Panels (**A**,**B**)), after 48 h of treatment with 20 ng/mL PDGF-BB or transfection of the SMCs with control mimics (MC) and mimic for miR-193a (M193a). After transfection, the cells were kept in starving media for 48 h prior to lysis. Total Akt and β-actin were used as loading controls. n = 3, * *p* < 0.05 compared to the respective control.

It is well-established that PDGF-BB drives cell growth in SMCs by activating Akt phosphorylation and, subsequently, upregulating the positive modulators of the cell cycle in the G1 phase such as cyclin-dependent kinase 4 (CDK4), Cyclin D1, retinoblastoma-protein (RB) phosphorylation, Cyclin E; downregulating the negative regulator of cell cycle p27; and driving cells into the S phase via the stimulation of DNA polymerase (Proliferating cell nuclear antigen; PCNA). Hence, we assessed whether miR-193a mimics modulate the effects of PDGF-BB on the expression of these key signaling proteins. As shown in Figure 12, the treatment of SMCs with either PDGF-BB or miR-193a (transfection) induced Akt phosphorylation by 7478 ± 2584% and 129 ± 58% (compared to their controls), respectively (*p* < 0.05 compared to untreated control, C; or mimic control, MC; n = 3).

**Figure 13:**
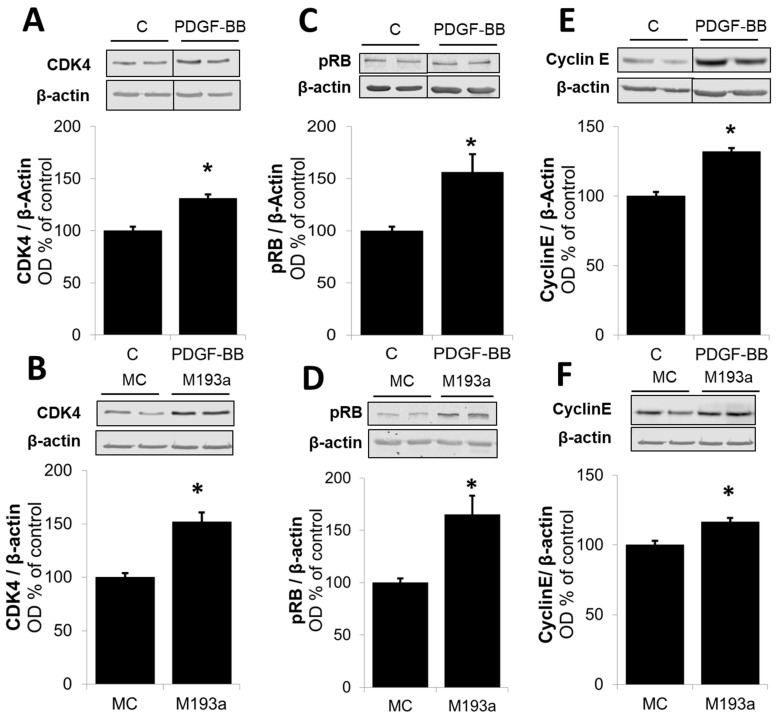
Overexpression of miR-193a promotes G1-to-S-phase progression. Representative Western blots and bar graphs of cyclin-dependent kinase (CDK4; Panels (**A**,**B**)), phosphorylated Retinoblastoma protein (pRB; Panels (**C**,**D**)), and Cyclin E (Panels (**E**,**F**)) after 48 h of treatment with 20 ng/mL PDGF-BB or transfection of the SMCs with control mimics (MC) and mimic for miR-193a (M193a). After transfection, the cells were kept in starving media for 48 h prior to lysis. n = 3, * *p* < 0.05 compared to the respective control.

**Figure 14:**
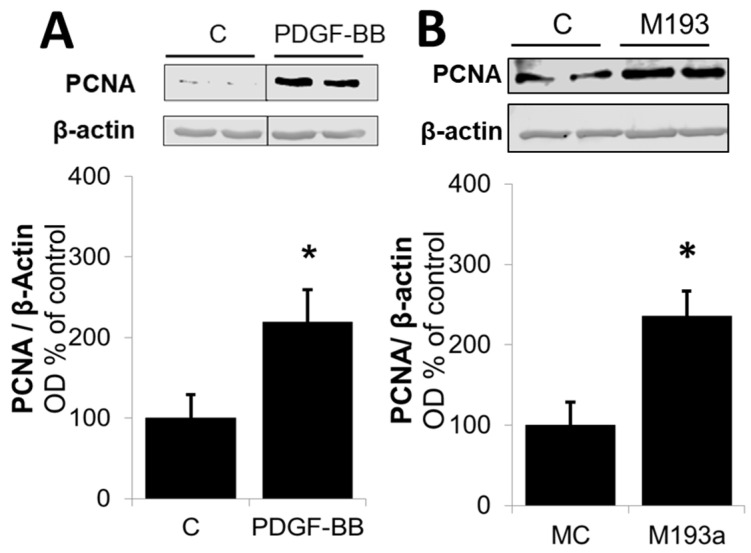
Overexpression of miR-193a promotes G1-to-S-phase progression. Representative Western blots and bar graphs of proliferating cell nuclear antigen (PCNA) expression after 48 h of treatment with 20 ng/mL PDGF-BB (Panel (**A**)) or transfection of the SMCs with control mimics (MC) and mimic for miR-193a (M193a; Panel (**B**)). After transfection, the cells were kept in starving media for 48 h prior to lysis. n = 3, * *p* < 0.05 compared to the respective control.

Next, we compared the effects on the positive regulators of the G1 cell cycle. As shown in Figure 13, and consistent with our observation for Cyclin D1 in Figure 6, both PDGF-BB and miR-193a induced the expression of the following: CDK4 by 31 ± 4% and 52 ± 9% (compared to their matched controls), respectively; pRB by 56 ± 17% and 65 ± 18% (compared to their matched controls), respectively; and Cyclin E by 32 ± 3% and 17 ± 3% (compared to their matched controls), respectively (Figure 13). Moreover, both PDGF-BB and miR-193a mimic induced the expression of PCNA in SMCs by 119 ± 40% and 136 ± 31% (compared to their matched controls), respectively; and confirming their stimulatory effects on DNA-polymerase and the activation of SMCs in the S phase (Figure 14).

**Figure 15:**
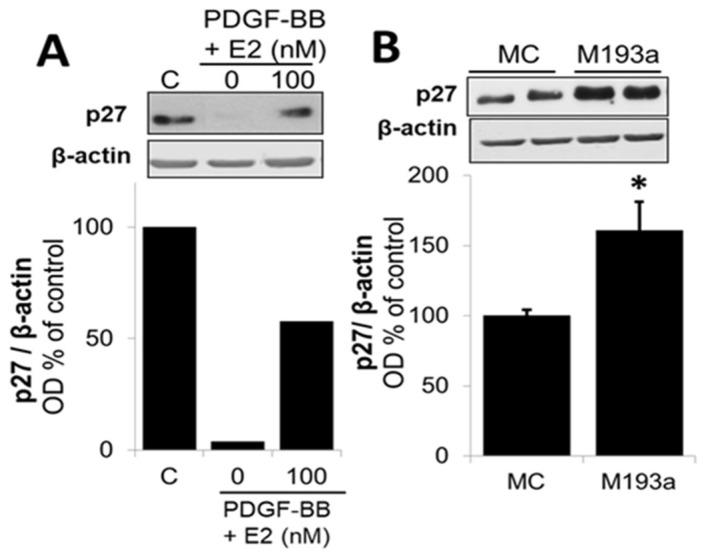
Differential modulation of p27 by PDGF-BB and miR-193a (M193a). Representative Western blots and bar graphs of p27 expression after 48 h of treatment with 20 ng/mL PDGF-BB in presence or absence of E2 (100 nM; Panel (**A**)), or transfection of the SMCs with control mimics (MC) and mimic for miR-193a (M193a; Panel (**B**)). After transfection, the cells were kept in starving media for 48 h prior to lysis. n = 3, * *p* < 0.05 compared to the respective control.

With regard to the impact of PDGF-BB and miR-193a on p27, a negative regulator of the cell cycle in the G1/2 phase, treatment with PDGF-BB downregulated its expression, whereas miR-193a induced p27 expression by 60 ± 21% compared to control (Figure 15). These observations suggest that PDGF-BB and miR-193a may differentially regulate p27 to induce SMC growth. Indeed, it has been shown that p27 can mediate both pro- and anti-oncogenic actions [11]. It is known that p27 binds and inhibits cyclin-dependent kinase (CDK) to arrest the cell cycle. Moreover, p27 also regulates other processes including cell migration and development independent of its CDK inhibitory action. Hence, the growth effects of PDGF-BB and miR-193a on SMCs via p27 may differ, and further in-depth studies will be required. Interestingly, treatment with E2 abrogated the inhibitory effects of PDGF-BB on p27 expression, suggesting that its inhibitory actions may, in part, be mediated via p27 upregulation.

### 3.7. MiRNA-193a Antimir Prevents Cuff-Induced Femoral Artery Lumen Occlusion and Wall Thickening

**Figure 16:**
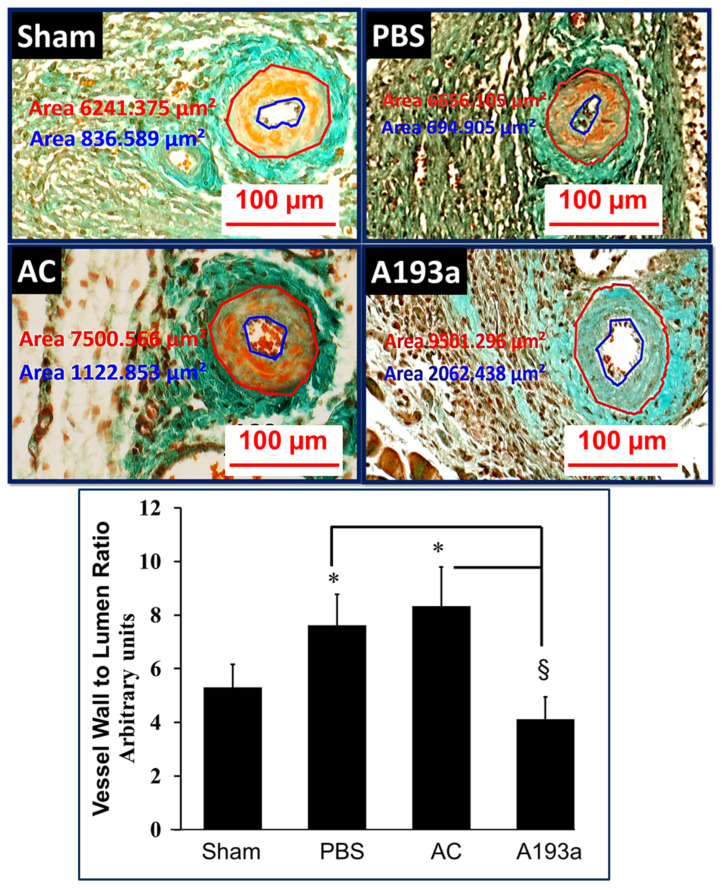
Representative photomicrographs (top panel) and bar graph (bottom panel) depicting the inhibitory actions of miR-193a-3p antimir on cuff-induced vascular remodeling femoral artery sections. Phosphate-buffered saline (PBS); antimir control (AC); and miR-193-3p antimir (A193a). Compared to animals in sham group, cuff placement increased vessel-wall-to-lumen ratio in animals receiving phosphate-buffered saline (PBS) and antimir control (AC), but not in animals receiving antisense miR-193a (A193a). Importantly, compared to animals receiving PBS or AC, cuff-induced vessel-wall-to-lumen ratio was significantly reduced in animals treated with A193a. * *p* < 0.05 vs. sham; ^§^ *p* < 0.05 vs. AC and PBS.

Since PDGF-BB induces miR-193a expression in SMCs and M193a mimics the effects of PDGF-BB on SMC growth, together with the observation that the pro-growth actions of PDGF-BB are abrogated by miR-193a antimir, we hypothesized that mi-R193a antimir may protect against vascular occlusion. Since PDGF-BB has been shown to play a prominent role in the cuff-induced occlusion of the femoral artery, we assessed the impact of miR-193a antimir on cuff-induced occlusion. As shown in Figure 16, the delivery of A193a in pluronic acid prevented the cuff-induced occlusion/narrowing of the femoral lumen and decreased the vessel-wall-to-lumen ratio (representative photomicrographs and bar graph) and prevented lumen narrowing. Compared to mice treated with AC, the lumen area was increased from 778 µm^2^ in AC to 2775 µm^2^ in A193a-treated mice (*p* < 0.05; n = 4).

## 4. Discussion

The protective effects of estrogens in the cardiovascular system are well-established and include the inhibition of vascular SMC growth (proliferation and migration), and the prevention of injury-induced intimal hyperplasia; however, the mechanisms involved remain unclear. The role of miRNAs in the vasculature has been extensively studied [16,18,19]. Estrogens have been associated with miRNA expression in different cell types [20] and several studies have shown that E2-regulated miRNAs potentially mediate their actions in different tissues, including the vascular system [12,31]. Hence, the aim of this study was to assess the role of miRNAs in mediating the protective effects of E2 in vascular SMCs. Our findings provide the first evidence that E2 abrogates PDGF-BB-induced cell proliferation and migration by downregulating miR-193-3p formation. Moreover, miR-193-3p is a key positive regulator of SMC growth, whereas its antimir has growth inhibitory actions in SMCs and cuff-induced vascular remodeling.

First, we assessed the expression of six miRNAs (miR-100, miR-193a, miR-203., miR-221, miR-222, and miR-638) after the treatment of SMCs with E2. These miRNAs were selected following literature research. MiR-100 attenuates murine vascular SMC proliferation and migration by suppressing the mammalian target of rapamycin (mTOR) [49], while its regulation by E2 has not been studied yet. MiR-203 specifically inhibits mouse aortic SMC proliferation [26] and attenuates the PDGF-stimulated proliferation and extracellular signal-related kinases 1 and 2 (ERK1/2) phosphorylation in human airway SMCs [50]. Moreover, there is evidence that the anti-mitogenic actions of E2 in murine vascular SMCs are mediated by the upregulation of miR-203 [26]. MiR-638 is highly expressed in quiescent cells and abrogates PDGF-BB-induced Cyclin D1 expression, and cell proliferation and migration in human aortic SMCs [51]. Moreover, miR-638 has been shown to be downregulated by E2 and to mediate its inhibitory effects on pericyte migration [52], making it an interesting target. In contrast to the described antimitogenic miRNAs, miR-221 and miR-222 play a prominent role in the vascular tissue by modulating key signaling pathways related to vascular SMC de-differentiation, proliferation, migration, and inflammation [40,53,54,55]. Interestingly, there is evidence that E2 downregulates miR-221 and miR-222 expression in MCF-7 cells [56,57]. Regarding miR-193a, it was recently shown that miR-193-3p controls the phenotype switch in SMCs [45]. Moreover, we recently observed that E2 downregulates it in EPCs (unpublished data) and provided evidence that miR-193a inhibits growth and vasculogenesis in ECs [31] (see accompanying article [38]); however, the role of miR-193a in vascular SMCs has not yet been elucidated. Since E2 inhibits PDGF-BB-induced SMCs growth [58] and injury-induced vascular remodeling which involves PDGF-BB [59], we elected to use PDGF-BB to assess whether it mediates its mitogenic actions in SMCs via miR-193-3p and whether E2 mediates its antimitogenic actions in SMCs, modulating miR-193a.

We found that treatment with PDGF-BB significantly induces the expression of multiple miRNAs (miR-100, miR-221, and miR-222) including miR-193a. Moreover, E2 inhibited the PDGF-BB-induced expression of these miRNAs in a concentration-dependent manner. However, the other tested miRNAs, miR-203 and miR-638, were unaffected by both PDGF-BB and E2 treatment. In contrast to the results from the literature [26], we did not observe any change in miR-203 in SMCs treated with E2. Similarly, we could not confirm the findings from Li et al. showing the PDGF-BB-dependent downregulation of miR-638 [51]. Among the regulated miRNAs, we found that PDGF-BB upregulated miR-100, suggesting a positive role for this miRNA in proliferation processes. This contrasts with the findings of Grundmann et al. [49], who demonstrated reduced vascular SMC growth in miR-100 overexpressing murine vascular SMCs. A potential reason for the discordant findings might be the difference in cell type and the species of origin. Indeed, miR-638 was studied in human aortic SMCs, whereas miR-203 and miR-100 were analyzed in murine cells. Regarding miR221/222, we found that these miRNAs are induced by PDGF-BB and downregulated by E2 in vascular SMCs. Our results are similar to the previous reports showing that miR221/222 are induced by PDGF-BB in vascular SMCs and mediate PDGF-induced phenotypic changes [60]. Finally, we provide the first association of miR-193-3p and vascular SMCs. Nevertheless, miR-193a-5p has been shown to inhibit vascular SMC phenotypic transformation in aortic aneurysm [61], and there is also evidence for an anti-mitogenic role for miR-193a in different cancer cells [62,63]. In contrast, we found that miR-193-3p is upregulated by PGDF-BB, suggesting that it might be involved in the promotion of cell growth. In agreement with this hypothesis, Wang et al. supports a pro-proliferative role for miR-193a in bone mesenchymal stem cells [64], indicating that it may play contrasting dual functions depending on the tissue or cell type where it is expressed. Moreover, miR-193a-5p exhibits a dual role in various cancer types, functioning both as an oncogene and a tumor suppressor [65]. Therefore, it is feasible that miR-193a positively contributes to SMC proliferation and migration and exhibits differential effects in vascular cells.

In summary, we discovered that the expression of miR-100, miR-193a, miR-221, and miR-222 is induced by PDGF-BB, suggesting a growth-promoting role in vascular SMCs. Moreover, we provide the first evidence that these miRNAs are downregulated by E2, indicating that they could be potential mediators of the inhibitory actions of E2 in SMCs. As miR-193a mediated the effects of E2 in vascular EC, we further assessed its role in SMCs.

Next, we examined whether E2 inhibits miR-193a transcription by assessing the pri-miR-193a levels, as E2 is known to interact with its nuclear receptors and directly regulate gene expression, including miRNA expression, by regulating their transcription [12]. Surprisingly, we found that treatment with PDGF-BB decreased pri-miR-193a expression at every tested time point, although mature miRNA levels are induced by PDGF-BB. Interestingly, E2 reversed the effects of PDGF-BB on pri-miRNA, showing increased expression compared to PDGF-BB, recalling its inhibitory action on PDGF-BB-induced mature miRNA levels. Since E2 reversed both the inhibitory effects of PDGF-BB on pri-miRNA expression and stimulatory effects of PDGF-BB on mature miRNAs, we speculate that PDGF-BB stimulates the maturation process of these miRNAs, depleting the pri-miRNA levels in sustenance for a higher mature miRNA expression, and that E2 restrains it. Nevertheless, further research is necessary in order to test our assumption and clarify the mechanisms by which PDGF-BB induces miR-193a.

Regarding E2, it has been shown to deregulate key miRNA biosynthesis pathway genes [66] and there is evidence for DiGeorge syndrome critical region 8 (DGCR8) upregulation and DICER1 and AGO-2 downregulation in E2-treated EC, suggesting that estrogens regulate the endothelial miRNA production machinery [12]. Moreover, the E2–ER interaction plays a role in Drosha and Dicer function [16,48]. In support of our hypothesis, we found that E2 downregulates PDGF-induced miR-193a via ERα, suggesting that E2–ERα may block PDGF-induced miRNA procession. Taken together, we provide evidence that E2 does not inhibit miR-193a transcription in SMCs. Moreover, based on the recent findings that E2–ERα plays a role in Drosha and Dicer function, we postulate that E2 downregulates PDGF-induced miR-193a by inhibiting its procession from the primary transcript to mature functional miRNA.

The second aim of the study was to assess the role of miR-193a in SMC function. Therefore, we transfected the cells with miR-193a mimics and antimirs to, respectively, upregulate and inhibit the miRNA expression. Interestingly, we found that miR-193a mimics induced cell numbers, BrdU-incorporation, wound closure, and Cyclin D1 expression similarly to PDGF-BB, while blocking miR-193a resulted in decreased cell growth and migration, similar to the effect of E2 in SMCs. These findings, together with the fact that miR-193a expression is stimulated by PDGF-BB, suggests that miR-193a mediates the mitogenic actions of PDGF-BB on SMCs. Although the association between PDGF-BB and miR-193a has not specifically been studied, PDGF-BB regulates the expression of some of its known targets through miRNA alterations in cancer cells [67] and miRNAs are also well-known to be involved in PDGF-BB signaling pathways in the vasculature [68]. For example, miR-221 is transcriptionally induced upon PDFG-BB treatment in vascular SMCs, leading to the downregulation of targets which are critical for the PDGF-mediated induction of cell proliferation [60]. Therefore, here, we provide evidence for a novel mechanism by which PDGF-BB induces SMC growth.

Changes in cell proliferation and migration may be influenced by cell death. The role of miR-193a in vascular SMC survival has not been investigated; nevertheless, miR-193a has previously been shown to regulate apoptosis in several tumors, including melanoma, hepatocellular carcinoma, acute myeloid leukemia, and breast and prostate cancer [69]. However, we could not detect any changes in cell viability by miR-193a by PI staining.

We and others have previously shown that miR-193a has an inhibitory effect in the vascular endothelium [31,44]. In the current study, however, we show a differential pro-proliferative action on SMCs. This differential role can be explained by the fact that miRNAs can have a contradictory effect on different cells. Indeed, there is extensive evidence that miR-193a acts as a tumor suppressor in cancers, such as colorectal, breast, and gastric cancer [63], while it has also been previously shown to increase cell proliferation and migration in renal cell carcinoma [70], osteosarcoma, and pancreatic carcinoma [71], suggesting a cell-type-specific function.

Interestingly, restoring miR-193a levels after E2 treatment completely reversed the inhibitory action of E2 on cell number and wound closure, suggesting that miR-193a is involved in mediating E2 actions on PDGF-BB-induced vascular remodeling and that its downregulation in SMCs may mediate the vascular protective effects of E2. These findings are similar to our previous results, showing that E2-induced vasculogenesis is mediated by miR-193a downregulation in ECs (see accompanying article [38]), suggesting that miR-193a might be responsible for the differential protective effects of E2 in vascular cells. However, these assumptions should be further investigated. Taken together, we provide the first evidence that miR-193a expression is upregulated by PDGF-BB and positively participates in the regulation of vascular SMC proliferation and migration. Moreover, we hypothesize that the regulation of miR-193a by PDGF-BB and E2 mediates their actions on the proliferation and migration of SMCs.

Since we found that miR-193a regulation mediated the protective actions of E2 in vascular SMCs, we investigated the role of ERs and found that ERα mediates the downregulation of miR-193a by E2. Although there is ample evidence for a role of ERα in mediating the anti-mitogenic actions of estrogens in vascular SMC [72,73], which ER is responsible for the beneficial effect of E2 in the vascular system is still intensely debated. In support of our findings that ERα is required for the downregulation of miR-193a, Di Leva et al. reported that E2–ERα mediates the repression of miR-221/222 in MCF-7 cells [56] and Zhao et al. demonstrated that ERα mediates miR-203 upregulation by E2 in vascular SMCs and that miR-203 participates in the anti-mitogenic actions of E2 in these cells [26]. In summary, here, we provide the first evidence that ERα is involved in miR-193a regulation, hence strengthening our hypothesis that E2 can be mediating its regulatory effects on vascular SMCs by miR-193a downregulation. However, we did not further investigate the molecular mechanisms by which E2–ERα inhibit miR-193a expression; thus, they remain unknown.

E2 metabolites are known to play an important role in the vascular system, and 2ME, an endogenous metabolite of E2 with no affinity for ERs, is known to mediate the protective action of E2 by blocking vascular SMC growth and injury-induced neointima formation through the double blockade of the cell cycle [11]. Therefore, we, next, assessed the effects of 2ME on miR-193a. Interestingly, we found that, in contrast to ECs (see accompanying article [38]), 2ME inhibits miR-193a expression in SMCs in a concentration-dependent manner. Since 2ME is known to inhibit SMC as well as EC growth [10], together with the fact that miR-193a inhibits EC growth and induces SMC growth, we speculate that 2ME may mediate its anti-proliferative actions in ECs and SMCs by modulating miR-193a expression. Our findings suggest that miRNA-193a is differentially regulated by 2ME in ECs and SMCs and mediates its anti-mitogenic actions. Interestingly, in contrast to 2ME, E2 induced EC growth and upregulated miR-193a expression, whereas, in SMCs, E2 downregulated miR-193a expression and inhibited SMC growth similar to 2ME. Taken together, our findings suggest that E2 and 2-ME modulate miR-193a levels to mediate their mitogenic or growth inhibitory actions in ECs and SMCs.

With our finding that 2-ME modulates miR-193a levels to mediate its anti-angiogenic actions in ECs and growth inhibitory actions in SMCs, together with the fact that 2ME induces anti-carcinogenic actions and has been postulated as a potential non-carcinogenic substitute of E2 hormone therapy, it is tempting to postulate that miR-193a antimir may have a therapeutic potential to protect against SMC-induced vascular occlusion without increasing the risk for cancer. The use of the miR-193a mimic or antimir may also circumvent the potential limitation of the rapid clearance of 2ME in vivo [10]. Interestingly, a synthetic mimic for miR-193a has recently been shown to induce tumor-suppressive actions [74]. However, the therapeutic potential of the miR-193a mimic or antimir as a 2ME substitute in preventing vascular occlusion and pathophysiologies linked to angiogenesis, for example, tumor progression/metastasis, needs further investigation.

With regard to the molecular mechanisms involved, it is well-known that PDGF-BB induces cell growth in SMCs by activating Akt phosphorylation and, subsequently, upregulating the positive modulators of the cell cycle in the G1 phase (CDK4, Cyclin D1, RB phosphorylation, and Cyclin E), downregulating the negative regulator of cell cycle p27, and driving cells into the S phase via the stimulation of the DNA polymerase (PCNA). Moreover, E2 inhibits SMC proliferation, at least partially, by the downregulation of the cell cycle progression from the G1 to S phase, thus blocking the pro-proliferative effects of PDGF-BB.

Here, we provide evidence that E2 blocks PDGF-BB-induced Cyclin D1 expression. Furthermore, the suppression of the cyclin-dependent kinase inhibitor p27 by PDGF-BB is reversed by E2, suggesting that E2 causes the G1 arrest of vascular SMCs via the inhibition of Cyclin D1 and the induction of p27. In support of our findings, Takahashi et al. have shown that E2 inhibits PDGF-BB-stimulated vascular SMC proliferation by reducing PDGF-BB-induced Cyclin D1 expression and inhibiting the phosphorylation of RB [75]. Indeed, the modulation of key G1-phase cell-cycle regulators is an important mechanism by which E2 affects proliferation. In fact, several studies have shown that E2 controls the Cyclin D1, c-Myc, CDK2, CDK4, and CDK inhibitors in MCF-7 breast cancer cells, thus promoting the progression from the G1 to S phase of the cell cycle [76,77].

Since we postulate that miR-193a downregulation may mediate the anti-mitogenic actions of E2 on PDGF-induced SMCs and E2 arrests vascular SMCs in the G1 phase of the cell cycle, we further investigated whether miR-193a is acting on the key signaling mechanisms as PDGF/E2 and if it mimics the effects of PDGF-BB on these molecules. Indeed, we found that miR-193a stimulates Akt phosphorylation and induces early cell cycle regulator Cyclin D1, CDK4, Cyclin E, and PCNA expression, as well as RB phosphorylation, thus reinforcing our hypothesis that miR-193a promotes the cell cycle progression from the G1 to S phase.

Various studies demonstrate that miR-193a inhibits the G1/S transition and proliferation of melanoma and breast cancer cells by targeting cell cycle proteins, including CDKs, cyclins, and p27 [78,79,80]. However, we found that miR-193a induces the growth of SMCs by upregulating Cyclin D1, CDK4, pRB, Cyclin E, and PCNA, which indicates that miR-193 results in a positive regulation of the cell cycle progression, suggesting that miR-193a’s role may vary depending on the cell type. Although there is more evidence about the impaired cell cycle progression, we provide abundant evidence for a pro-proliferative role of miR-193a. Moreover, previous findings show that the upregulation of miR-193 promotes the proliferation of bone mesenchymal stem cells by inducing CDK2 expression [64], supporting our assumption that miR-193a might differentially regulate cell cycle components depending on its cellular function. Taken together, our findings show that miR-193a induces SMC growth through the promotion of cell cycle regulators. However, the underlying mechanisms involved need to be further elucidated.

The major function of D cyclins is to provide a link between mitogenic stimulus and the cell cycle machinery. Therefore, it is feasible that increased Cyclin D1 levels and the downstream cell cycle effectors are due to the induction of upstream signaling cascades regulating proliferation processes, such as the Ras/Raf/ERK and phosphatidylinositol 3-kinases (PI3K)/Akt signaling pathways. In the current study, we investigated the effects of miR-193a on the PI3K/Akt pathway and demonstrated that miR-193a increases Akt phosphorylation in SMCs. However, the stimulation of Akt phosphorylation is slight in comparison to the considerable increase after treatment with PDGF-BB, suggesting that this is not the main mechanism by which miR-193a induces vascular SMC proliferation and that the potent effects of PDGF-BB may be due to a combination of factors other than the stimulation of miR-193a. Indeed, in addition to the regulation of PI3K/Akt, the anti-mitogenic actions of E2 include the inhibition of Raf/Mek/Erk mitogen-activated protein kinase (MAPK) signaling, which plays a prominent role in proliferation. Because E2 inhibits SMC growth by abrogating MAPK phosphorylation [58], we speculate that miR-193a may contribute to vascular SMC growth also through the induction of this MAPK pathway. In support of our findings, miR-193a promotes fracture healing via the regulation of Phosphatase and Tensin homolog (PTEN) [81], a negative regulator of Akt. Moreover, it has been shown to directly target PTEN in renal cell carcinoma [82] and in an Alzheimer Disease model [83]. Therefore, it is possible that miR-193a increases PI3K/Akt signaling in SMCs through targeting its negative regulator PTEN. On the other hand, it indirectly inhibited the PI3K/Akt cascade in non-small-cancer cells [84] and we previously demonstrated that it inhibits Akt phosphorylation in vascular EC (see accompanying article [38]), suggesting that miR-193a differentially regulates the PI3K/Akt signaling pathway depending on the cell type and context.

We also investigated the effect of miR-193a on p27 expression, a negative regulator of the cell cycle in the G1/S phase. PDGF-BB has been extensively shown to downregulate p27 [85]; indeed, it consistently inhibited p27. Surprisingly, we found that miR-193a induced cell cycle inhibitor p27, which seems in contrast to its pro-proliferative action. The effect of miR-193a on p27 has solely been studied by Uhlmann et al. His group investigated the impact of different miRNAs, including miR-193a, in the epidermal growth factor receptor (EGFR)-driven cell cycle pathway and showed that miR-193a upregulates p27 expression in breast cancer cells [78]. However, in contrast to vascular SMCs, miR-193a exhibits anti-proliferative actions in breast cancers cells and also inhibits CDK4 and Cyclin D1, while these proteins were upregulated in our study. Therefore, we cannot compare our findings on increases in p27 levels with the findings in breast cancer cells, as the growth outcome was opposite.

To block cell cycle progression, p27 inhibits the phosphorylation of RB by cyclins, thus preventing their separation from RB, which prevents the transcription of genes required for G1/S transition, includes cyclins such as Cyclin A, Cyclin D, and Cyclin E [86]. The phosphorylation of RB is one of the most crucial steps regulating the progression of the cell cycle as it allows the release of E2F and the subsequent induction of genes required for DNA synthesis. Here, we demonstrate that, in spite of a high p27 expression, RB is phosphorylated and E2F is probably released, since we observed an increase in the Cyclin E and PCNA expression. These findings support the pro-proliferative role of miR-193a in SMCs and suggest that, although the p27 levels are high, the molecule may not be functionally active.

Interestingly, it has been shown that p27 can mediate both pro- and anti-oncogenic actions [87]. It is well-known that p27 binds and inhibits cyclin-CDK to arrest the cell cycle, thus blocking proliferation. On the other hand, p27 also regulates other processes including cell proliferation, migration, apoptosis, and development independent of its CDK-inhibitory action. Indeed, p27 concentration is regulated at different levels, including transcription, translation, protein stability, and degradation [88], while its function is modified by its subcellular localization [89,90]. Indeed, p27 may function as a tumor suppressor in the cellular nucleus while acting as an oncoprotein in the cytoplasm [89]. Depending on its phosphorylation, p27 is degraded in the nucleus or exported to the cytoplasm where it is either degraded or retained. In quiescent cells, the p27 levels are high and bound to CDK2 to ensure the complete inhibition of kinase activity. In proliferating cells, p27 needs to be inactivated in order to allow the Cyclin E-CDK2 complexes to actively phosphorylate RB and release E2F, thus allowing the transcription of genes important for DNA synthesis in the S phase. Upon mitogenic stimulation, the Ras and/or PI3K pathways are activated and trigger c-Myc and Akt to influence p27. c-Myc directly represses p27 expression, by promoting its degradation [91]. Akt directly regulates the expression of p27 through phosphorylation, which results in p27 redistribution and retention in the cytoplasm [92]. Since miR-193a induced Akt phosphorylation in our study, it is feasible that miR-193a increases p27 expression in the cytoplasm by preventing its suppression, thus promoting its proliferation promoting actions. However, this remains speculative and needs to be further investigated.

Taken together, in the current study, we observe that miR-193a positively regulates the cell cycle progression through the induction of Cyclin D1, CDK4, pRB, Cyclin E, and PCNA. However, the direct targets for the miRNA remain unclear. We speculate that miR-193a may target negative regulators of the cell cycle or the mitogenic signal transduction mechanism(s) upstream of Cyclin Ds. However, increasing evidence indicates that miRNAs not only repress their targets, but also induce their expression through the miRNA–promoter interaction depending on the particular cellular conditions, cell type, and context [93]. Hence, it is also feasible that miR-193a may act as an activator for its target genes in SMCs. However, further examinations are necessary in order to investigate this hypothesis.

**Figure 17:**
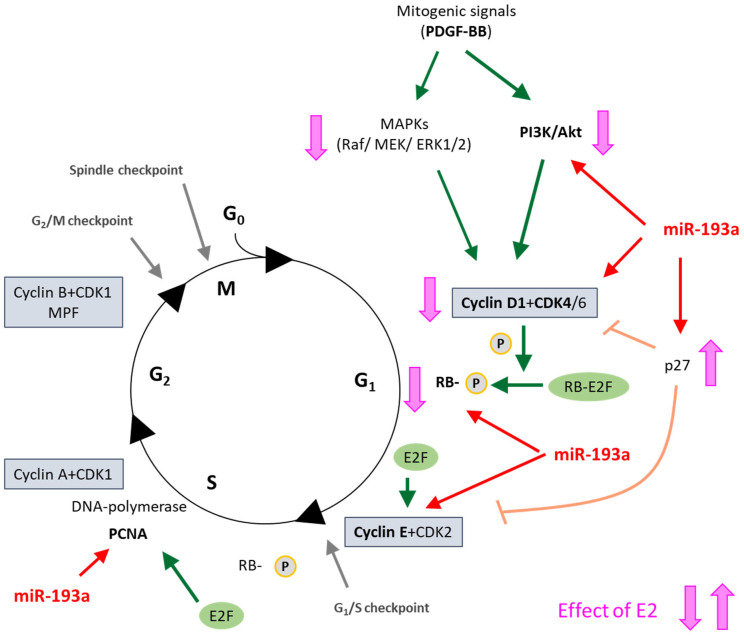
Summary of our findings in a simplified schematic representation of the cell cycle in vascular SMCs and its regulation by E2. Green arrows depict PDGF-BB action; Red arrows show MiR-193a action; and Pink-orange line show E2 action on the respective cell cycle regulators. G_0_ quiescence; G_1_, Gap 1; S, Synthesis; G_2_, Gap 2; M, Mitosis; PDGF-BB, Platelet-Derived Growth Factor BB; MAPK, Mitogen-Activated Protein Kinase; PI3K, Phosphoinositide 3-Kinase; CDK, Cyclin-Dependent Kinase; RB, Retinoblastoma protein; PCNA, Proliferating Cell Nuclear Antigen; MPF, Mitosis Promoting Factor.

In summary, we confirmed that E2 inhibits PDGF-BB-induced vascular SMC proliferation by blocking Cyclin D1 expression and upregulating the cell cycle inhibitor p27, suggesting that it inhibits the cell cycle progression from the G1 to S phase. Moreover, we provide insight into the molecular mechanisms by which miR-193a influences vascular SMC proliferation (Figure 17). Like PDGF-BB, the overexpression of the miRNA induces Akt phosphorylation, indicating the activation of the PI3K/Akt pathway, and promotes the cell cycle progression by inducing the expression of Cyclin D1, CDK4, and Cyclin E and increasing RB phosphorylation. However, in contrast to our expectations and the results from other researchers, p27 was induced. Nevertheless, we found that miR-193a induced PCNA expression and promoted DNA synthesis. Hence, it is likely that p27 upregulation is not associated with increased p27 activity. In conclusion, the mechanisms by which miR-193a regulates cell cycle progression needs to be further elucidated. A better understanding of how the regulation of these miRNAs by PDGF-BB and E2 to mediate their pro-proliferative and/or anti-mitogenic actions, respectively, in vascular SMCs, is required.

PDGF-BB plays an important role in injury-induced vascular remodeling [94] by increasing SMC growth and migration and promoting neointimal formation. Therefore, we finally investigated the role of miR-193a on vascular occlusion in animal models. Our findings show that the inhibition of miR-193a by its antimir prevents the cuff-induced occlusion and narrowing of the femoral lumen, suggesting that miR-193a inhibition by E2 may indeed be involved in the protective actions of E2 on the vasculature. Importantly, PDGF-BB plays a major role in cuff-induced vascular remodeling and lumen occlusion [35]; moreover, blocking the PDGF-BB receptor prevents injury-induced neointimal thickening [95]. The importance of PDGF-BB in mediating the vascular remodeling process during vascular injury is well-documented [96]. Together with the fact that E2 inhibits cuff-induced vascular occlusion [59] and cuff-induced remodeling is associated with an increase in PDGF-BB gene expression [35], it is tempting to speculate that E2 mediates its vascular protective actions by downregulating miR-193a formation. The fact that, similar to E2, miR-193a induced contrasting growth effects on SMCs and ECs and miR-193a antimir mimicked the effects of E2 suggests that it mediates the vascular protective actions of E2. However, our findings do not rule out the involvement of other miRNAs and mechanisms that may also work in parallel.

Our finding that E2 inhibits PDGF-BB-induced SMC growth by downregulating miR-193a-3p, together with the fact the miR-193a antimir prevents cuff-induced vascular occlusion in mice, suggests that miR-193a antimir may be of therapeutic relevance against vascular occlusion in humans. However, our in vitro findings may not fully translate in a complex in vivo setting where inter-individual differences in the presence of different growth factors, pathologies, and vascular cell biology may influence the inhibitory actions of miR-193a antimir. Importantly, the presence of other miRNAs as well as the stability and availability of miR-193a could influence its effects, in vivo. Moreover, reproducing the anti-mitogenic actions of E2 mediated via the downregulation of miR-193a in SMCs in vitro may depend on the expression of vascular ERα, in vivo. Hence, further in-depth studies are required in order to assess the feasibility of our in vitro observations in an in vivo setting.

## 5. Conclusions

In the present study, we found that miR-193a promotes vascular SMC growth and that mediates the mitogenic actions of PDGF-BB. We hypothesize and demonstrate that E2 mediates its protective effects by downregulating miR-193a and abrogating its pro-proliferative actions. Interestingly, in an accompanying paper [38]), we demonstrate that miR-193a inhibits vascular EC function and that E2 mediates its pro-angiogenic effects, in part, via the downregulation of this miR-193a. Our findings demonstrate that miR-193a antimir mimics the differential growth effects of E2 on SMCs and ECs. Although our findings suggest that E2 may mediate its vascular protective actions by modulating miR-193a, it also plays an important role in cell growth, migration, and the invasion of various tumor cells, acting both as a tumor suppressor or oncogene depending on the cancer cell type [62,63,64,65,66,67,68,69,70,71]. The fact that miR-193a differentially regulates distinct vascular cells such as EC and SMCs and the downregulation of miR-193a shows protective effects on both cell types makes this molecule a very interesting therapeutic target not just against cancer but also in the vascular system for the treatment of cardiovascular disorders and abnormal vascular remodeling. Indeed, the use of a miR-193a antimir could both limit vascular SMC abnormal growth and intimal hyperplasia, while restoring EC function and re-endothelization after vascular injury.

Limitations: Although our study demonstrates E2 mediates its anti-growth effects on SMCs by downregulating miR-193a, the participation of miRNAs cannot be ruled out. Moreover, whether growth factors other than PDGF-BB induce miR-193a and whether E2 blocks them similarly remain unknown and must be investigated. Finally, whether our observation that miR-193a-3p-antimir inhibits SMC growth and prevents cuff-induced vascular occlusion in mice can be translated/reproduced in the complex in vivo environment in humans remains questionable. Although promising, further studies are required in order to confirm the relevance of our findings in humans.

## Data Availability

All data supporting the findings of this study are available within the article or from the corresponding author upon reasonable request.
